# How Effective Are Biodiversity Conservation Payments in Mexico?

**DOI:** 10.1371/journal.pone.0119881

**Published:** 2015-03-25

**Authors:** Sébastien Costedoat, Esteve Corbera, Driss Ezzine-de-Blas, Jordi Honey-Rosés, Kathy Baylis, Miguel Angel Castillo-Santiago

**Affiliations:** 1 Institute of Environmental Science and Technology (ICTA), Universitat Autònoma de Barcelona, Barcelona, Spain; 2 Department of Economics and Economic History, Universitat Autònoma de Barcelona, Barcelona, Spain; 3 Center International en Recherche Agronomique pour le Développement (CIRAD), Montpellier, France; 4 School of Community and Regional Planning, University of British Columbia, Vancouver, British Columbia, Canada; 5 Agriculture and Consumer Economics Department, University of Illinois, Urbana, Illinois, United States of America; 6 Laboratorio de Análisis de Información Geográfica, El Colegio de la Frontera Sur, San Cristóbal de las Casas, Chiapas, Mexico; University of New South Wales, AUSTRALIA

## Abstract

We assess the additional forest cover protected by 13 rural communities located in the southern state of Chiapas, Mexico, as a result of the economic incentives received through the country's national program of payments for biodiversity conservation. We use spatially explicit data at the intra-community level to define a credible counterfactual of conservation outcomes. We use covariate-matching specifications associated with spatially explicit variables and difference-in-difference estimators to determine the treatment effect. We estimate that the additional conservation represents between 12 and 14.7 percent of forest area enrolled in the program in comparison to control areas. Despite this high degree of additionality, we also observe lack of compliance in some plots participating in the PES program. This lack of compliance casts doubt on the ability of payments alone to guarantee long-term additionality in context of high deforestation rates, even with an augmented program budget or extension of participation to communities not yet enrolled.

## Introduction

**Fig 1:**
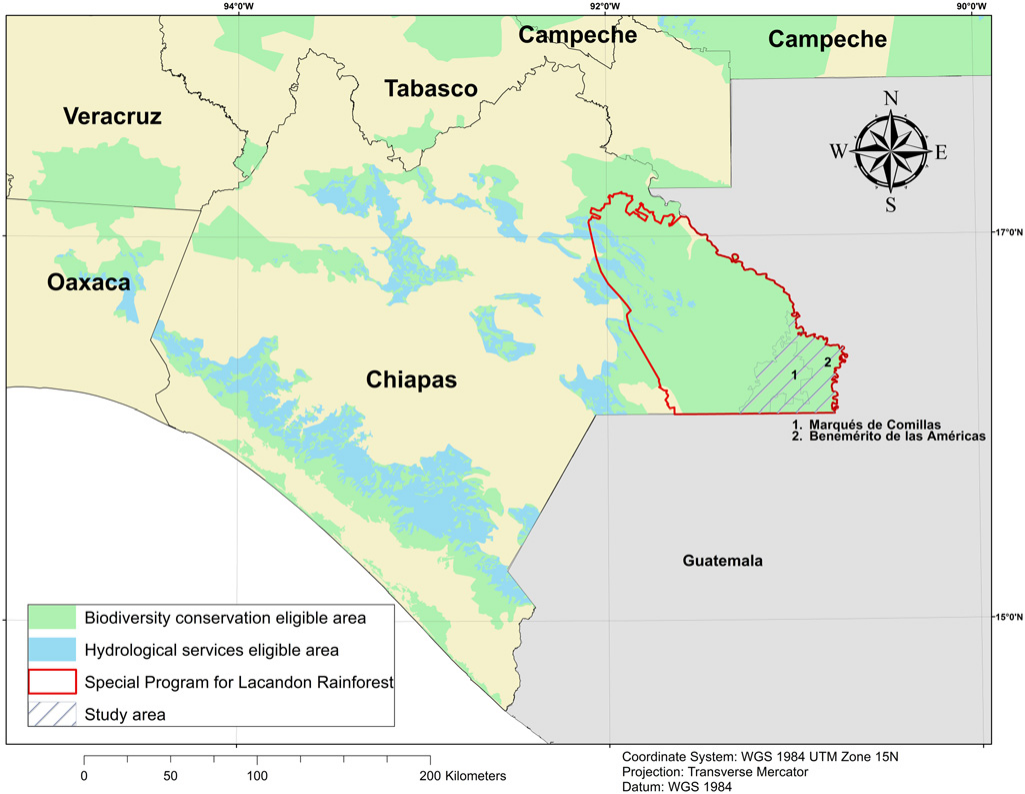
PES eligibility areas (2012) in Chiapas. The Special program for Lacandon rainforest run in parallel with the federal PES program. Source: CONAFOR for programs eligibility areas, INEGI for state and municipal boundaries and GADM for international boundaries.

**Fig 2:**
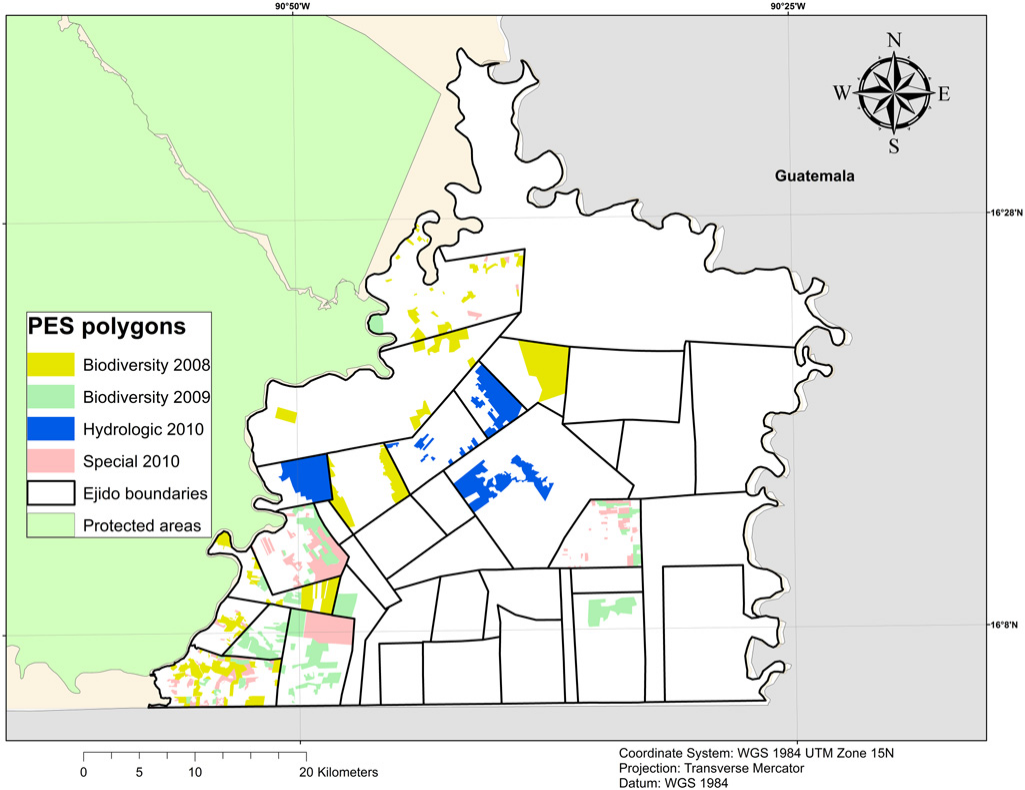
PES polygons in the study area. Each parcel under PES contract is geo-referenced by technical service providers. This map includes the polygons that were included in PES between 2008 and 2010 in our study area. We do not include PES polygons which are currently under contract. Source: CONAFOR for PES polygons, CONABIO for protected areas, INEGI for municipal limits and GADM for international boundaries. *Ejido* boundaries have been elaborated by the authors and are only presented as indicative.

Payment for Environmental or Ecosystem Services (PES) programs have become increasingly important for biodiversity and ecosystems conservation worldwide, including in developing countries [1–3]. Proof of their environmental effectiveness in contributing to avoided deforestation or the provision of specific ecosystem services is just emerging [3–5]. This article provides insight on the effectiveness of Mexico’s national program of payments for biodiversity conservation, focusing on avoided forest loss as a proxy outcome. The analysis focuses on two municipalities in the state of Chiapas which were selected due to their high deforestation rates [6] and the increasing support received from government and NGOs to conserve their tropical forests (Fig. 1). Between 2005 and 2013, 19 out of the 37 communities located in these municipalities had received at least one PES contract (Fig. 2).

PES programs are voluntary transactions between at least one buyer and at least one seller, in which payments are transferred in exchange for the preservation of a well-defined environmental service [2,7]. PES are conditional economic incentives structured around a formal legal contract or a participant parties' agreement [8,9], and they have been implemented in various institutional, economic and environmental contexts [2,10–12]. PES schemes have targeted a range of ecosystem services, including watershed regulation, carbon sequestration, or multiple bundled services through specific land-use measures supporting biodiversity conservation and multi-functional landscapes. PES schemes differ in their underlying institutional frameworks and implementation scales: while some are built upon complex institutions that articulate the quantification and exchange of well-defined services, such as payments for carbon sequestration under the Kyoto Protocol's Clean Development Mechanism global market or wetland banking and riverine restoration in the United States, other schemes rely on flexible, or project-based institutional frameworks that do not or cannot use provision of strict measures of ecosystem services as their output metric due to methodological and cost constraints [2,13–17].

PES programs are seen as a promising tool to conserve tropical forest in many developing countries [2,5]. Mexico, along with Costa Rica, has been a pioneer in the implementation of PES programs at national and sub-national levels. Mexico has lost about half of its forests over the last 50 years, but remains one of the most forested countries in the world [18]. The state of Chiapas alone contributed to 12% of national forest loss between 1993 and 2007 despite representing only 3.7% of the country’s territory [6]. Mexico's government-funded *Pago por Servicios Ambientales Hidrológicos* (PSAH) scheme is managed by the National Forestry Commission (CONAFOR) and has been operational since 2003 [2]. In 2004, CONAFOR developed a second PES program aimed at paying landowners for the provision of carbon sequestration services, biodiversity conservation or for the development of agroforestry systems (PSA-CABSA). After several reforms, both initiatives were merged into a broader forestry program currently known as PRONAFOR [19,20]. The current PES program only provides incentives for two modalities of ecosystem services provision, namely hydrological services and biodiversity conservation. Despite its name, the program in its current form does not directly monitor the provision of these ecosystem services and it is focused exclusively on supporting the conservation of standing forests. In parallel, CONAFOR also co-sponsors supplementary local PES schemes at the local level and other initiatives linked to REDD+ early actions [21–23].

Mexico's PES programs are open to both privately and community owned lands. Around 70% of the country's forests are owned by *ejidos* and indigenous communities [24]. *Ejidos* are a type of collective tenure recognized after the 1910 Mexican revolution and are generally comprised of both family-run-*de facto* private- land plots and common resource management areas. In turn, indigenous communities are recognized by the State as a form of social property existing prior to the revolution, with subsequently much older customary arrangements for the management of the resource commons. PES programs indistinguishably support landowners, *ejidos* and communities and reward them annually over a renewable five-year period, during which the recipients are mandated to develop forest management and conservation activities on selected lands. Payment levels have historically differed across modalities, with biodiversity conservation forest parcels receiving on average lower payments per hectare than hydrologic services (Table 1). More than 2.6 million hectares have been included in the program (2% of the national forested area were under PES contract in 2007, year of greatest program extension), which translates into a budget of around 450 million USD [25]. To date, the biodiversity modality represents roughly 25% of the total land area allocated to PES programs.

**Table 1:** PES payment level by cohort of beneficiaries in selected municipalities.

Cohort	2008	2009	2010	2011	2010	2011	2012
PES Modality	Biodiversity	Biodiversity	Hydrologic	Biodiversity	Special	Special	Special
N beneficiaries	7	8	4	1	7	3	2
Area under PES (ha)	6367	4253	4174	1823	3324	1,187	595
Annual payment by ha	394	411	550	550	1000	1000	1000
Equivalent in USD	30.19	31.49	42.15	42.15	76.63	76.63	76.63

The modality corresponds to the ecosystem service recognized in the municipalities according to the definition of eligible areas of the cohort of interest. Since 2010, special program for Lacandon rainforest and federal PES program are operating simultaneously in the municipalities of our study area. The area under PES corresponds to the total area put under PES for the modality of interest in our study area. The payment level by modality is defined in the procedural rules published annually. The annual payment by hectare is expressed in Mexican pesos and do not evolve during the five-years of the contract. The equivalent in USD is calculated using the exchange rate for the 1^st^ of January 2014 (13.05 Pesos/USD). Source: List of participants are published annually on CONAFOR website.

The environmental additionality of PES programs is not easily measurable because the reference baseline is hypothetical and thus not observable. Recent development of quasi-experimental methods in environmental sciences allows researchers to estimate robust counterfactuals based on empirical data [3,9,10,11]. Available empirical studies, however, are ambiguous about the effectiveness of PES schemes [3,26]. Evidence from Mexico and Costa Rica shows that their respective PSAH and PES national programs have had a limited impact on avoided deforestation because, in their early years, payments were allocated to areas with low deforestation risk [27,28]. However, analyses performed at sub-national level in regions with higher deforestation have found more significant positive impacts on avoided deforestation [25,29,30].

The contribution of our analysis is twofold. First, we focus on the effectiveness of Mexico's PES biodiversity conservation modality because, to our knowledge, it has been overlooked to date. Most empirical studies of PES effectiveness have focused on the hydrological services scheme [25,21,31]. Second, we perform our analysis comparing grid cells spread over two municipalities, which involved identifying suitable control groups with good statistical properties in a geographically constrained area that is subject to high deforestation pressure. As exogenous units of analysis, grid cells help address the lack of tenure data at the plot level and also allow us to integrate information from different geographical and administrative scales. We define the size of the spatially explicit grid cells to capture the variation of physical characteristics within each *ejido*.

The analysis shows that deforestation is proceeding apace in the two selected municipalities, with lower rates in communities participating in the PES program. The implementation of the PES program has resulted in significant environmental additionality, indicating a much larger effect than in other studies that analyze the country’s PSAH program over a larger geographical area [25,27]. Our different specifications show that, on average, additional conservation represents between 12 and 14.7 percent of the area enrolled under PES. The following sections present our materials and methods, describe our results in detail and discuss them in light of debates around PES contribution to environmental conservation, leakage effects, and the role of payments in changing land managers' behavior.

## Materials and Methods

### Analytical framework

Impact assessment approaches are becoming a standard in environmental program evaluations [3,27,32]. In particular, quasi-experimental approaches explicitly address the selection bias inherent in each program based on voluntary or selective participation [3–5,26]. These approaches are derived from Rubin’s potential outcome framework and are assumed to propose credible causal inference about the impacts of an intervention [26,33]. This framework notes that it is impossible to observe the outcome of a unit in both treated and non-treated states; therefore, we cannot observe the effect of the treatment on this unit (i.e. Fundamental Problem of Causal Inference) [33]. Nevertheless, we can estimate the Average Treatment Effect (ATE) by using the outcome of a group of non-treated units as a counterfactual for the outcome of a group of treated units [33,34].

Among the methods existing to estimate the treatment effect, the difference-in-difference (DID) estimator replicates a natural experiment in which we compare the outcome of treated and non-treated unit groups over at least two periods, i.e. before and after the implementation of the treatment [5,32,34]. The non-treated group is used as a control, and we can subtract the outcome gains of the non-treated from the gains of the treated. The advantage of this method is that it removes the bias from permanent differences between the two groups as well as it neutralizes common time trends that are not-related with the treatment [34]. The method is nevertheless based on strong assumptions, such as parallel trends in the absence of treatment or independence of outcomes between time periods and within groups [26,34,35]. These assumptions are not testable if we only have two time-periods and two groups [34].

Matching methods reinforce the credibility of DID estimations because they select similar units among the non-treated units to construct the control group according to a "distance" measure. Among the variety of "distance" indexes proposed in the literature, the Mahalanobis distance takes into account the inverse of the covariance matrix for all the covariates chosen by the researcher [34]. This approach means that each treated unit is paired with its "nearest neighbor(s)" according to the value of the chosen covariates. Although matching is often considered as a method to estimate the treatment effect in itself, some have also proposed to use it to pre-process data in order to obtain a balanced sample with sufficient overlap in the distribution of the two groups [34,36].

Even with precise identification strategies, there is a risk of missing important factors-e.g. particular unobservable characteristics- that can determine the treatment group and the outcome of interest [34]. In our analysis, we have limited this problem by examining program implementation in a geographically limited area where territories have relatively similar historical trends, land use patterns, and potentially similar access to information and to infrastructure.

### Study area

Our study area encompasses the municipalities of *Marqués de Comillas* and *Benemérito de las Américas*, which include 23 and 14 *ejidos*, respectively (Fig. 1). The municipal limits have been chosen as study area boundaries because in this case they capture political frontier—between Mexico and Guatemala- and natural barrier- the Lacantun River separate the municipalities from the protected rainforest of the Montes Azules Biosphere reserve (Fig. 2).

In the 1970’s, the study area held large tracts of rainforest but as of today less than half of the original cover remains today. Over this period, these municipalities have had one of the highest deforestation rates in the country [6]. Most forest loss in the past was driven by government settlement policies, which brought large numbers of landless farmers from around the country to Chiapas between 1970 and the early 1990s [37]. Present threats to forests are linked to agricultural and pasture expansion [6], which are activities that are encouraged by government programs, in addition to a flat topography [37]. Livestock raising has been the main livelihood activity until this last decade, when new productive activities promoted by private actors, such as African oil palm cultivation-now grown in 26 of the 37 *ejidos*-, have gained prominence [6,37]. Both governmental and non-governmental conservation policies and projects began to be implemented during the 1990s, focused on controlling slash and burn agriculture, and illegal timber logging [6,26]. However, limited funding, monitoring and enforcement by both state and federal governments, have contributed to their limited success [6].

PES programs have recently emerged in the two selected municipalities as a complementary conservation incentive in a context of increasing tensions between land-use change and a growing number of conservation initiatives. For example, our two selected municipalities are located within a biological corridor established in 2007 to connect various Biosphere reserves in the southern part of Mexico [37,38], and in addition to other areas located in the states of Jalisco and of the Yucatán peninsula, they belong to one of the three key target early action areas for Reducing Emissions from Deforestation, forest Degradation and enhancing carbon stocks (REDD+). In Chiapas, early actions are structured around the Special Program for the Lacandon rainforest (*Programa Especial de la Selva Lacandona* or PESL, for its Spanish acronym), which is implemented in eight municipalities that still have standing rainforest. This special program includes a PES mechanism specifically designed to address local drivers of deforestation and forest degradation, among other incentives for sustainable use and rainforest conservation.

### Participation in Mexico's PES programs

CONAFOR annually publishes the eligible areas and selection criteria for participating in the federal PES programs [18,20,28]. Privately and community owned lands can be included in PES contracts [21,25]. In the case of *ejidos*, the decision to participate is a collective choice decided by the *ejido* assembly, which is the most important decision-making body made up of all right holders. The parcels included in program funding applications can involve all or a portion of or the community’s commonly managed forests, or selected forested parcels controlled by households. Applicants can hire technical service providers to develop their application proposal and payment-targeted parcels are geo-referenced as polygons. Once an application is approved by CONAFOR, the proponent(s) must design a forest management plan on the contracted polygons, which is also developed with service providers' support.

In our selected municipalities, the first PES contract was signed in 2005 but the program gained momentum in 2008, when seven *ejidos* were granted a contract for biodiversity payments (Fig. 2). As noted, 19 *ejidos* are now involved in the program. The remaining 18 *ejido*s have declined or have been unable to participate and, among these, there are four *ejido*s lacking administrative pre-requisites to become PES eligible. According to CONAFOR officials, others do not participate because they do not own sufficient primary forest or their forests have already been divided among too many families and converted into other land uses. The rest have simply refused to participate because they are not interested or do not have the ability to make a collective decision in this regard. As the program is voluntary, forest owners can decide not to participate in the program regardless of the level of forest cover found in their properties.

### Data and unit of analysis

We would like to be able to compare forest conservation outcomes within PES polygons with similar un-treated polygons. However, the polygons are generated exclusively for the needs of the PES program, therefore there is no database of un-treated polygons. Nevertheless, information systems are well developed in Mexico [31] and the National Agricultural Registry (RAN) provides *ejidos* and communities information about land tenure. Because polygons and *ejido* boundaries are geo-referenced, we were able to build a spatially explicit database to select units that were to be used as controls.

**Fig 3:**
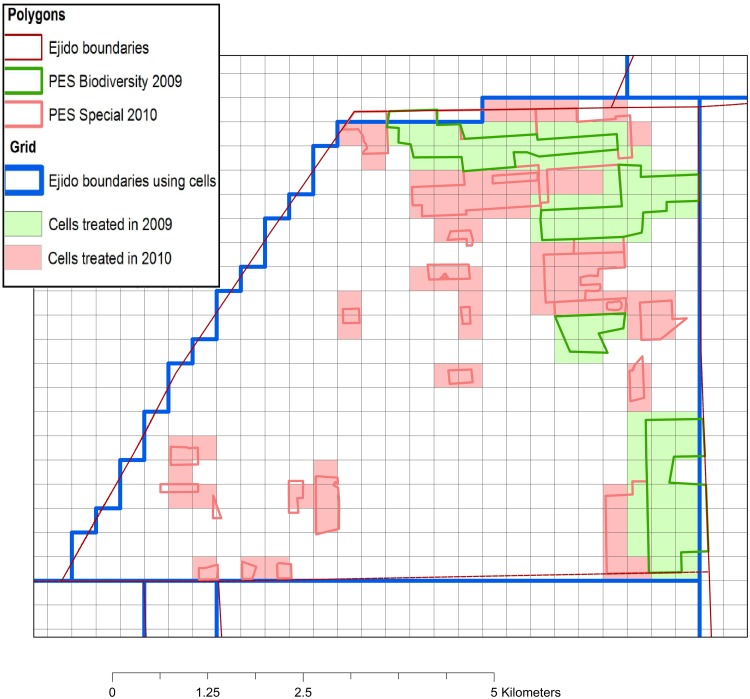
Transformation occurring by the use of grid cells as unit of analysis. The construction of our unit of analysis is based on an exogenous regular grid. Due to the irregularity of PES polygons and *ejido* boundaries, the shapes can be altered. Here is the case of an *ejido*, which has received two PES contracts, one starting in 2009 and one started 2010. Source: CONAFOR for PES polygons, *ejido* boundaries are authors’ elaboration.

**Fig 4:**
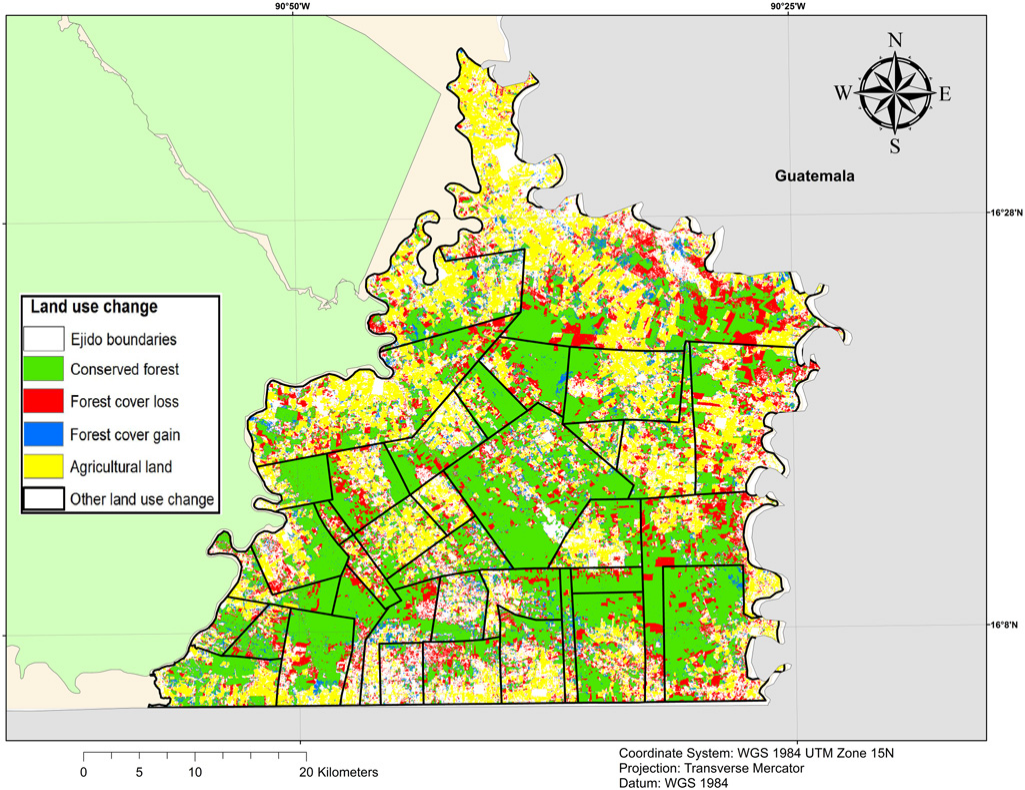
Land use change (2007–2013). Land use change map is obtained by the classification of two Spot 5 images. The classification is supervised with a maximum-likelihood process completed with manual correction in areas with spectral confusion. This map present only land use changes related to forest cover and agricultural land. Source: INEGI for municipal limits, GADM for international boundaries. *Ejido* boundaries are authors’ elaboration and land use change map has been elaborated from Spot 5 satellite images using ERDAS IMAGINE 9.2.

Our data sources are published in different formats. Physical and land use information are defined at a pixel level while program and administrative data are available as polygons. We defined a spatially explicit grid composed of regular squares of 10 hectares—approximately a side length of 316 m—to capture different scales of information. This size allowed us to account for the precision of spatially explicit data while being below the minimum PES eligible area. Each grid cell represented an exogenous unit of analysis, which does not correspond strictly to decision-making units [3,29] (Fig. 3). However, it permitted us to generate a spatially explicit database that captures a variety of land uses within each *ejido*. In addition, within a treated *ejido*, we distinguished between areas treated by the PES program and unenrolled or un-treated parcels. The main limitation of the use of cells for comparison purposes is a distortion at the boundary between two communities: such boundaries are irregular and a grid cell may overlap with two communities. Therefore, to categorize a cell as being in one community or another we used the community in which the cell’s centroid resided to determine the community information associated with that cell. Visual interpretation of community boundaries indicated that, in the municipalities selected, there was no evidence of any forest encroachment from one community into another, as community boundaries were easily distinguishable by contrasting land uses (Fig. 4).

We also used grid cells to capture additional information from other datasets, in particular: i) biophysical features of the landscape (land use, elevation, slope, soil characteristics) and ii) administrative and census data that is assumed to be common across all cells in the same community. We acquired Spot 5 satellite images (10-m resolution) for 2007 and 2013 to detect land use changes (Fig. 4). For each year, we generated two maps comparing land cover to capture changes before and after the implementation of PES contracts for our first cohort of interest. We considered eight land-use classes: mature forest, disturbed forest, other vegetation, cultivated lands, rivers, wetlands, urbanized area and undefined area (e.g. roads, clouds or shadows). These classes were a simplification of the classification used by [6]. Our classification, however, had the advantage of making a neat distinction between different vegetative cover, and it particularly distinguished mature from disturbed forests in regeneration, as the former are the target of the PES program. In the study are, some forests are still very dense and a variety of native species can be found while other show manifest signs of regrowth. Nevertheless, we were not able to differentiate between natural regeneration and the introduction of new species.

**Fig 5:**
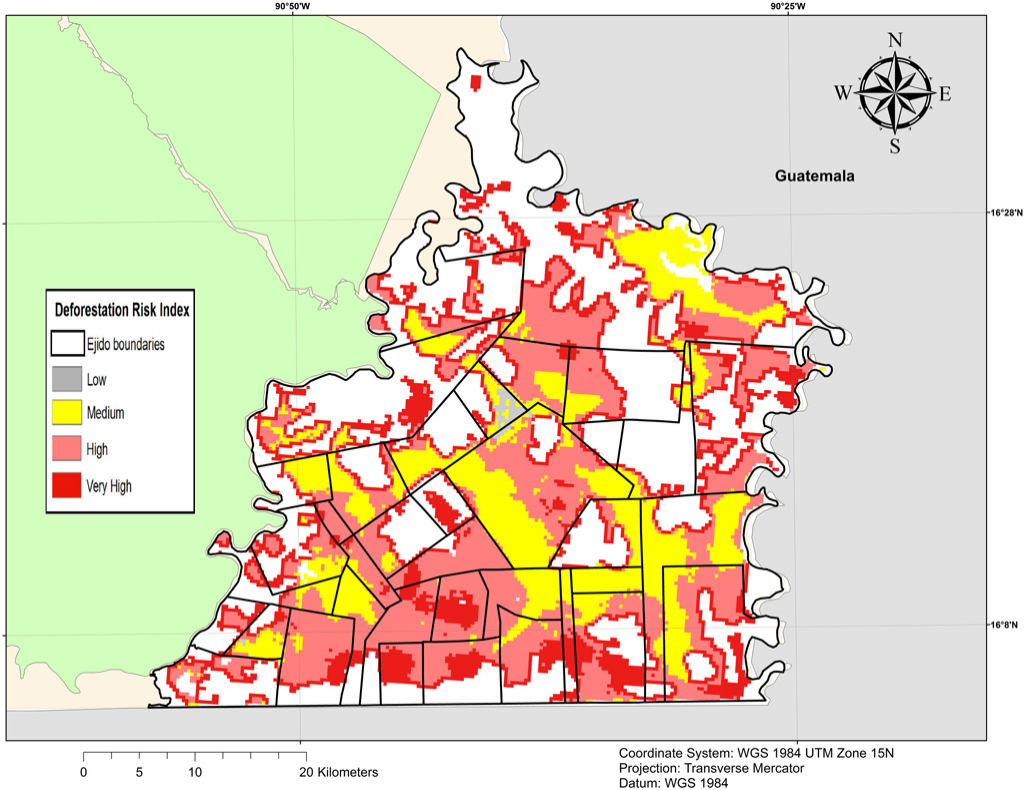
Deforestation risk index. The deforestation risk index has been developed by the National Institute of Ecology and Climate Change (INECC) and it is a predictive probability taking into account economic pressures likely to cause deforestation at pixel level. Source: INECC for Deforestation risk index, INEGI for municipal limits and GADM for international boundaries. *Ejido* boundaries are authors’ elaboration.

For each cell, we calculated total forest cover in 2007 and 2013. Our dependent variable took into account the area of forest conserved between the two periods (in ha) instead of a binary variable indicating forest conservation or not at pixel level. Continuous variables capture more information than binary variables, especially where selective logging is frequent [29]. The National Institute of Ecology and Climate Change (INECC) has developed a Deforestation Risk Index to enhance PES programs targeting across the country [25] (Fig. 5). This index predicts the risk of forest loss based on an econometric model calibrated with data related to deforestation drivers from 2000 to 2007. The index is presented in the form of a probability of deforestation (from 0 to 1) computed at pixel level. We aggregated theses pixels to obtain an average deforestation probability at cell level. We completed our data with a digital elevation model (DEM) elaborated by the National Institute of Statistics and Geography (INEGI), available for all Mexican territory. We used Arcgis to compute altitude and slope and aggregated the information at cell level.

We also added information from a geomorphologic database on soil quality [39]. Soil quality indicates land suitability for agriculture since it influences relative land productivity. Soil classes include steep slopes, alluvial plain, structural plain, lake plain, eroded land and river valley. Alluvial plains are the most fertile class. Nevertheless, the impact of soil quality on deforestation is ambiguous: indeed, a more productive soil type could increase deforestation to maximize profits but it can also help to conserve forests by facilitating more intensive agricultural practices that require less land. For each cell, we computed spatially lagged variables for all the available physical characteristics (elevation, slope, forest cover and deforestation risk). We used Geoda to calculate these variables using a queen contiguity matrix (W matrix) [29]. We also relied on census data from INEGI at the *ejido* level. The census provides other demographic information and an asset-based marginality index used as a proxy for poverty levels. We used data from the 2005 census as pre-program values for *ejidos* and data from the 2010 census as post-program values. Consequently, all cells of an *ejido* have the same values for these census variables.

### Defining treatment areas

We focused on communities that joined the PES program in 2008 or 2009, since these were the largest participating cohorts of the study area. For a cell to be considered treated, at least 0.5 ha had to overlap with the PES polygon in our less restrictive classification, and 8 in our most restrictive classification. We excluded cells with lands belonging to another PES cohort because these cells were already treated and would have introduced bias. When a cell overlapped with two or more PES polygons within the same *ejido*, we considered the cell as treated by the earlier PES contract.

### Identification strategy

Several authors such as [25] or [30] pre-process data to reduce the sample to treated and un-treated units that have similar observable characteristics, and to remove possible bias that can affect the measurement of program effectiveness. We suspect two sources of possible selection bias, one at the parcel level and the other at the *ejido* level. In particular, we performed three matching specifications using covariate matching with Mahalanobis distance: two were limited to matching over physical characteristics of cells (specifications A and B) and the third included socio-economic and demographic information at *ejido* level (specification C).

Specification A compared parcels treated in 2008 and 2009 to matched non-treated parcels within *ejidos* that received PES. We matched across biophysical characteristics: forest area in 2007, elevation, slope, deforestation risk index and the spatial lags for each of the four covariates. We also added an exact matching on the dominant soil in the cells, which served both as an index of relative soil productivity and as a way to control for the geographic location of the cells. Indeed, visual interpretation of the region's geomorphological map suggested that alluvial soils border rivers and protected areas while other soils are more present in remote areas. Our set of physical variables was assumed to capture opportunity costs at parcel levels. Because the control group included only parcels of *ejidos* treated in 2008 or 2009, we were neutralizing the influence of unobservable characteristics at *ejido* level such as decision-making processes and motivations. We present the covariate balance for all observable characteristics (Table 2, second column).

**Table 2:** Covariate balance in our three matching specifications.

	Treated		Control with matching on physical characteristics in treated ejidos (A)				Control with matching on physical characteristics in non-treated ejidos (B)				Control with matching on physical and socioeconomic characteristics in non-treated ejidos (C)			
	n = 1413		n = 805				n = 1111				n = 761			
ATT			1.11				1.1				0.9			
	Mean	SE	Mean	SE	% bias	p-val.	Mean	SE	% bias	p-val.	Mean	SE	% bias	p-val.
**Physical characteristics**														
Conserved forest (ha)	7.67	0.08	5.73	0.12	59	0	6.14	0.11	48.8	0	6.03	0.13	49.7	0
Forest area 2007 (ha)	8.43	0.06	7.78	0.09	20.8	0	8.14	0.07	9.3	0	7.9	0.09	16.8	0
Elevation (m)	175.04	0.58	176.06	0.76	-4.1	0.28	174.13	0.65	3.5	0.31	178.06	0.68	-12.1	0
Slope (%)	1.89	0.06	1.81	0.07	4.3	0.4	1.54	0.05	19	0	1.6	0.05	15.8	0
Deforestation risk	0.07	0	0.08	0	-7.8	0.05	0.07	0	2.2	0.53	0.07	0	0.5	0.89
**Socioeconomic characteristics**														
*Ejido* size (ha)	5211.8	85.3	6675.2	141.13	-16.3	0	10153	285.72	-54.9	0	5125.1	106.38	1	0.54
Years since *ejido* foundation	36.18	0.24	38.22	0.35	-20.8	0	34.1	0.28	21.2	0	33.11	0.31	31.4	0
Distance to city (km)	253.41	0.47	252.99	0.68	2.7	0.61	252.16	0.44	8.1	0.06	255.69	0.62	-15	0
Marginality index in 2005	0.11	0.62	0.15	0.02	9.7	0.03	0.39	0.02	-45.5	0	0.21	0.02	-15.5	0
Population in 2005	484.35	12.43	706.59	21.43	-11.7	0	1362.9	60.23	-46.2	0	537.78	15.93	-2.8	0.01
Active population (%)	0.52	0	0.51	0	-6.9	0.11	0.5	0	56.2	0	0.51	0	29.5	0
**Spatially lagged characteristics**														
Conserved forest (ha)	6.58	0.07	5.12	0.1	53.9	0	5.47	0.09	40.9	0	5.43	0.1	42.7	0
Forest area 2007 (ha)	7.44	0.06	6.74	0.08	26.2	0	7.13	0.07	11.7	0	6.93	0.08	19	0
Elevation (m)	174.71	0.55	175.89	0.73	-4.8	0.2	173.94	0.63	3.2	0.35	178.17	0.66	-14.1	0
Slope (%)	1.84	0.05	1.77	0.05	4.6	0.37	1.53	0.04	21.8	0	1.61	0.04	16.1	0
Deforestation risk	0.07	0	0.07	0	-9.9	0.02	0.07	0	1.8	0.61	0.06	0	6.2	0.11

We provide an estimation of the Average Treatment Effect of the Treated (ATT) for our three specifications. The first specification (A) preprocesses data based on a 1:1 matching on Mahalanobis distance with replacement. The potential control group is limited to ejidos that have parcels treated in 2008 or 2009. Covariate matching is based on forest cover in 2007, elevation, slope deforestation risk and their respective spatial lags. We add an exact matching characterizing the dominant type of soil in the cell. Specification B follows the same procedure but the potential control group is limited to ejidos which have not received any PES program. The last control specification (C) is obtained by a two-step procedure. We first match non-treated ejidos to treated ones using social and administrative variables (years since ejido foundation, ejido size, numbers of right holders, distance to nearest city, total population, proportion of active population and marginality index). We perform another matching 1:1 on Mahalanobis distance with replacement using the same procedure than in the first control group, adding an exact matching taking into account the pairs of ejidos previously defined. We make the comparison of group mean and standard error (SE) between treated and defined control groups. We only considers cells that had at least 1 ha of forest in 2007. A cell is considered as treated if it overlap (more than 2 ha) a PES polygon in 2008 or 2009. We also compare variable distribution between treated and the three respective control groups using two statistics. We use the P-value of mean-comparison test and the standardized bias difference in percentage. Standardized difference is the difference in percentage in average covariate values, divided by the square root of the sum of variances for both groups ([34]). W is the spatially lagged value using a queen contiguity matrix (average value of the eight cells that share a boundary with the cells of interest).

However, we suspect specification A may be biased because each *ejido* decided to enroll some parcels in the program and not others, and this determination was probably driven by unobservable characteristics related to opportunity costs. Consequently, we would expect un-enrolled forest parcels to be more likely deforested because they potentially had higher opportunity costs and *ejido* members could feel there was an implicit right to deforest because they did not receive incentives for conservation. Therefore, we ran the same matching specification but limited the potential control group to forested cells of non-treated cells *ejidos* (specification B). In this case, non-treated *ejido*s correspond to those that had never received a PES contract between 2005 and 2012.

Our third specification, specification C, takes into account the socio-economic and biophysical characteristics of cells. We developed a two-step matching and identified pairs of comparable *ejido*s using some of the variables proposed by [40]. For each treated *ejido*, we identified the most similar non-treated *ejido* in terms of socio-economic and demographic characteristics. In doing so, we used the following variables: distance to the nearest city, years since ejido creation, size, population with property rights, and other demographic information (i.e. total population, proportion of people between 15 and 64 years-old, number of people who have been in secondary school, and marginality index). The heterogeneity of *ejido* characteristics is very important, so only, eight non-treated *ejido*s were retained in our control group. The comparison of conservation outcomes between treated and matched *ejidos* would not be sufficient to measure program impacts. We then performed covariate matching with physical variables defined in the first specification. Therefore, for each treated parcel of a treated *ejido*, matched cells were more physically similar inside the more similar non-treated *ejido* (Table 2, fourth column).

We used the matching specifications to measure the Average Treatment Effect on the Treated (ATT), which corresponds to the average impact of the program on forest conservation for units that have received the program. In all our matching specifications, we employed a bias-adjustment procedure as proposed by [41] and we used both mean-comparison and standardized bias statistic to assess covariate balance [42]. In each case, we performed sensitivity analysis by discarding the worst 5% match according to Mahalanobis distance, by using two matches instead of one and by varying the minimum forest size (0.1 or 1 hectare). We also provide information about the sensitivity of our results to the thresholds used in the construction of our database. The first threshold is the minimum forest area chosen to distinguish a forested cell from a non-forest: we compare the results of a variation from a minimum forest area of 1 ha to an area of 0.1 ha. Our second threshold is related to the definition of treated cells. We consider different overlaps between a cell and a PES polygon (0.5, 2 and 8 hectares). The variation of this threshold either conserves or eliminates cells located at the boundaries between enrolled and un-enrolled forest parcels.

To test the robustness of each specification, we used a difference-in-difference (DID) estimator to control for time invariant factors that jointly affected control and treated units. We estimated the DID with i) an Ordinary Least Square (OLS) model and with ii) a Panel Fixed-Effects (FE) model. Our OLS specification contained our set of physical and socio-economic variables likely to have an influence on forest conservation independently of PES payments [35]. Because we only had two time periods, a FE model could not take into account eventual serial correlation. Nevertheless, we used panel variables that we considered good proxies for eventual change in decision-making capabilities to account for variations in social and demographic characteristics at *ejido* level. These variables related to the evolution of total population, marginality index, proportion of active population (between 14 and 65 years-old) and proportion of people with secondary education.

## Results

### Deforestation risk

Our results show that, on average, non-treated parcels in treated *ejidos* have a higher deforestation risk than treated parcels (respectively 0.074 against 0.066) (Table 3, line E and F). This is consistent with the fact that the deforestation risk index is a proxy for opportunity costs. Indeed, forests with low opportunity costs are more likely to be placed under the program by *ejidos*' right-holders because the payment received to conserve is higher than the expected profitability of alternative land uses on the allocated lands. We also observe that the average deforestation risk in non-participant *ejidos* is similar to that in treated parcels (Table 3, line E and I).

**Table 3:** Deforestation risk and forest cover loss by cohorts of participants and non-participants.

Parcels		Total area (ha)	Average Risk index	Forest cover in 2007 (ha)	Forest cover in 2013 (ha)	Forest loss (ha)	Forest loss (%)	Percentage of loss in study area	Deforestation rate (%)
PES parcels	Since 2005 (A)	3130	0.048	2790.48	2675.26	115.23	4.13	0.4	-0.84
	Since 2008 (B)	9570	0.075	6868.59	6375.91	492.68	7.17	1.71	-1.48
	Since 2009 (C)	7330	0.067	5038.38	4450.85	587.54	11.66	2.03	-2.45
	Since 2010 (D)	12550	0.065	8846.23	7782.29	1063.94	12.03	3.69	-2.53
Participant *ejidos*	Subtotal PES parcels (E)	32580	0.066	23543.68	21284.3	2259.38	9.6	7.83	-2
	Subtotal non PES parcels (F)	46540	0.074	31834.69	21285.99	10548.7	33.14	36.54	-7.73
	Subtotal *ejidos* treated (G)	79120	0.071	55378.37	42570.29	12808.08	23.13	44.36	-5.12
Non-participant	Subtotal *ejidos* non treated (I)	67640	0.067	42474.13	26410.31	16063.82	37.82	55.64	-9.07
Total		146760	0.065	97852.5	68980.59	28871.91	29.51	100	-6.75

We calculate the predicted deforestation risk index and the occurred deforestation for different groups of parcels. First, we compare cohorts of PES plots distinguishing the year of first involvement into a PES contract. Second, we aggregate these data and compare treated and non-treated plots within participant ejidos. Finally, we provide information for non-participant ejidos.

We refined our analysis for treated parcels by decomposing our results by year of first entry into PES. The first parcels to enter the PES program in 2005 had a very low deforestation risk. This finding is consistent with observations made by [21,27] and with the fact that the use of the deforestation risk index to select eligible areas for payment under Mexico's PES programs was only introduced in 2007 [23,31]. We can therefore see that parcels entering the PES program in 2008 have a higher probability of deforestation (0.075 on average) than previous cohorts. As the stock of forest is limited, forests entering PES in later cohorts have a lower deforestation risk-although higher than for the 2005 cohort-, which, however, is similar to the average of non-treated parcels in treated *ejidos*.

### Deforestation rate

In 2007, roughly half of the total area of the two municipalities was covered in primary forest, for a total area of 97,852.5 hectares (table 3, third column). By 2013, total forest cover was reduced to 68,980.6 hectares, which represents an average annual deforestation rate of 6.75%. From the summary statistics, it appears that there is little relation between the predicted deforestation risk and actual deforestation. If the local conservation programs are successful, we would expect this outcome as conservation activities and payments target those parcels with significant deforestation risk, ideally translating into reduced forest cover loss on those same parcels. This suspicion is further supported by the fact that the majority of deforestation (55.64%) occurs in non-treated *ejidos*, which have lost 37.82% of primary forest cover between 2007 and 2013 (table 3, line I). However, non-treated parcels of treated *ejidos* also face a high deforestation risk but have lost relatively less forest over the same period. This observation can be interpreted from two opposite perspectives. On the one hand, PES participating *ejidos* might increase their conservation practices even on un-enrolled parcels as a positive spillover of the program. On the other hand, it might be that participant *ejidos* would have conserved more forests than non-participant *ejidos*, even in the absence of the program. This second reason would be consistent with unobservable characteristics associated with governance or preferences for conservation affecting community selection into the PES program.

Considering compliance with PES goals, we observe that 7.83% of forest loss in the study area has been lost within PES polygons, mainly due to selective logging. This means that the PES contract terms have not been completely enforced, which can in turn be explained by the fact that PES contracts prohibit land use change but do not have explicit criteria for forest cover. Therefore, it is indicative that selective logging is informally allowed and not sufficiently monitored and sanctioned by government officers, which might contribute to forest degradation even within polygon boundaries. It is worth highlighting that forest cover loss is more prominent in parcels recently contracted under PES. However, forest loss on these parcels should be interpreted cautiously since we do not know precisely if these losses occurred before, during, or after the PES contract for the parcels entering the program in 2010 and beyond came into force.

### PES additionality for 2008 and 2009 cohorts

We estimated the ATT for our three above-described matching specifications (specifications A, B and C) (table 2). In our first specification, the ATT is 1.11, which means that, if a cell is treated, 1.11 ha of that given cell have been additionally conserved in comparison with the correspondent control group. As we have 1413 treated cells, the average avoided forest loss is 1568.43 ha, which represent 14.7 percent of the area put under PES in 2008 and 2009. In our second specification, which only considers parcels in non-participating *ejidos* as control group, the additionality of the program is similar, at 1.10 ha per cell (14.6 percent of enrolled area). The similarity of these results can be an indication of the absence of leakage effect in our first specification.

The last specification, which includes socio-economic variables, provides an estimation of the ATT that is substantially lower than the estimate from the second specification by nearly 0.2 ha by cell (but the avoided deforestation still represent about 12 percent of the enrolled area). Thus, if we compare similar parcels in similar *ejidos*, the additionality is relatively lower that if we only compare forest parcels that are similar in terms of biophysical characteristics. These differences are probably due to the fact that socio-economic and institutional factors have an impact on success of the program at community level.

### Sensitivity Analysis

We performed sensitivity analysis over three dimensions. First, we considered how the magnitude of the ATT estimate changed in response to modifying the matching parameters. Second, we check if the ATT estimate was sensitive to the threshold we outlined for the definition of minimum forest cover and minimum overlap with PES polygons. As observed in table 4, it appears that our results are somewhat sensitive to these threshold assumptions. Last, we explore the effect of the matching specification. We choose two matches per treated observation instead of one and only keeping the best 95% matches. As noted above, we find that our results are sensitive to including community characteristics, which implies that these socio-economic variables capture differences in the propensity of communities to enroll in the program and differences in forest cover outcomes. In both cases, however, the ATT is significantly different from zero. The analysis of the matching specifications suggests that results are relatively sensitive to the threshold we used to determine minimum forest cover and minimum PES overlap. Holding constant the PES overlap area but reducing the minimum forest area from 1 ha to 0.1 ha appears to systematically reduce the program impact in all our specifications. This difference suggests that the PES program is less effective in the conservation of small forested areas than for more compacted areas. Alternatively, the smaller minimum forest area may add noise to our dependent variables, leading to an error in variables problem, potentially biasing our estimated treatment toward zero.

**Table 4:** Sensitivity of ATT to matching parameters and thresholds.

	Control with matching on physical characteristics in treated *ejidos* (A)					Control with matching on physical characteristics in non-treated *ejidos* (B)					Control with matching on physical and socioeconomic characteristics in non-treated *ejidos* (C)				
	Minimum forested area (ha)	0.1		1		Minimum forested area (ha)	0.1		1		Minimum forested area (ha)	0.1		1	
	Minimum PES overlap (ha)	0.5	2	2	8	Minimum PES overlap (ha)	0.5	2	2	8	Minimum PES overlap (ha)	0.5	2	2	8
	n treated	1,646	1413	1413	707	n treated	1,646	1413	1413	707	n treated	1,646	1413	1413	707
Match 1:1 (1)	n control	939	826	805	473	n control	1309	1120	1111	601	n control	888	793	761	435
	ATT	1.00	1.11	1.11	1.00	ATT	0.96	1.09	1.10	1.20	ATT	0.78	0.91	0.90	0.89
	Variables with bias <20%	9	9	9	9	Variables with bias <20%	7	8	10	10	Variables with bias <20%	10	10	12	10
Best matches(2)	n control	894	796	776	473	n control	1259	1086	1070	594	n control	859	773	742	431
	ATT	1.06	1.14	1.10	0.97	ATT	0.94	1.02	1.03	1.15	ATT	0.84	0.94	0.91	0.79
	Variables with bias <20%	12	12	11	9	Variables with bias <20%	7	7	7	10	Variables with bias <20%	9	10	11	12
Match 2:1(3)	n control	1486	1315	1273	783	n control	2291	1997	1953	1088	n control	1374	1237	1208	738
	ATT	1.09	1.17	1.21	1.04	ATT	0.98	1.11	1.13	1.19	ATT	0.76	0.89	0.87	0.82
	Variables with bias <20%	11	11	11	11	Variables with bias <20%	7	7	7	10	Variables with bias <20%	9	10	11	10

We provide an estimation of the Average Treatment effect on the Treated (ATT). We provide statistics on the performance of matching using the two specifications. We provide variations of results according to different matching parameters. (1) is a 1:1 matching based on Mahalanobis distance with replacement. (2) only considers the best 95% matches of the 1:1 matching. (3) corresponds to a 2:1 matching with replacement. We provide different results by varying the minimum forest area and the minimum overlap with PES polygons.

We check this result by analyzing how estimates of additionality resulting vary with changes in the minimum PES overlap. Our 8 ha threshold restricts the treated units to only those largely overlapping PES polygons and, when doing so, the additionality in all our specifications diminishes. We attribute this result to the fact that some cells imperfectly overlapping PES polygons were considered as treated with a less strict threshold but became considered as control now.

### Robustness analysis

Finally, we performed additional estimations to assess the robustness of the previous estimation of program impacts (Table 5). In our sample, results from both OLS and FE models give a significant estimation of program impact. In both estimations, the estimated additionality is higher than with simple matching specifications. This result implies that either the time-invariant parcel characteristics or common time trends bias the treatment results downward.

**Table 5:** Robustness of specifications using difference-in-difference estimation.

Matching DID								
Matching on physical characteristics (1)			Matching on physical characteristics (2)			Matching on physical and social characteristics (3)		
	DID	SE					DID	SE
PES overlap>2 ha	1.28***	0.17	PES overlap>2 ha	1.24***	0.15	PES overlap>2 ha	1.1***	0.1693211

Matching DID with Fixed effects								
Matching on physical characteristics (4)			Matching on physical characteristics (5)			Matching on physical and social characteristics (6)		
	DID	SE					DID	SE
PES overlap>2 ha	1.27***	0.09	PES overlap>2 ha	1.05***	0.09	PES overlap>2 ha	1.13***	0.0973134

*** p-value< 0.01.

We provide results of various Difference-in-Difference (DID) estimations. (1),(2) and (3) correspond to OLS estimation of DID for each of our matching specification. We estimate the equation by adding control variables likely to explain conservation outcomes (elevation, slope, years since ejido foundation, ejido size, proportion of commons area, total population, proportion of active population, proportion of people with secondary education, number of right holders, distance to nearest city and marginality index. (4), (5) and (6) estimate using a DID in panel version using a fixed-effect. We add variables in panel version (marginality index, population, proportion of active population and proportion of population with secondary education).

## Discussion

Our results show that Mexico's PES program for biodiversity conservation has been effective in enrolling areas that generally show high deforestation risk. We also have shown that on average, treated parcels in recent cohorts and un-enrolled parcels have similar deforestation risk, which suggests that participation in PES cannot be explained only by land opportunity costs but, as other studies have suggested, also by factors related to collective decision-making and local governance [43]. The program has led to additional forest cover protection in comparison to what we would have expected in the absence of payments. This finding is consistent with the existence of a positive relationship between deforestation trends and the eventual environmental additionality of a PES program [23,24], although the level of additionality found is much higher than those observed in other impact evaluation studies of similar PES programs in Mexico [25,29].

The program's additionality diminishes to around 20% when taking into account socio-economic variables. Given the small number of observations, however, it has not been possible to determine the relative contribution of leakage effects to such diminished additionality. Robustness analysis confirms additionality but variations are found when testing for different classification thresholds for determining land use units comparison groups with respect to the size of the forest parcel and the level of overlap with the PES polygons. Lower additionality in PES lands with smaller forests suggests additionality could be lower in already fragmented forests surrounding agricultural or pasture lands. Further analysis is needed to confirm this relation but it raises doubts about the possibility to maintain additionality for current cohorts under contract. Indeed, in 2008, the minimum area that *ejidos* could enroll in programs was 20 ha, but in recent years the complementary Special Program for the Lacandon rainforest allows this minimum area to be split between several parcels.

The analysis also reveals that additionality can be achieved despite low compliance levels in some PES areas (i.e. plots under PES lands have been losing around 10% of forest cover during the contract). We argue that the lack of compliance can be explained by constrained forest governance at collective or household levels coupled with deforestation inertia, as well as by the government's insufficient enforcement of program rules. Regarding the former, it is important to highlight that non-compliant PES areas can encompass three types of tenure arrangements, namely: *de jure* commonly held and managed land; *de jure* commonly held land but *de facto* divided by the assembly and household managed; and *de jure* household-owned land. Additionally, we observed that un-enrolled parcels of treated *ejidos* have been less deforested than parcels of non-treated *ejidos* despite a higher deforestation risk index for the former can imply the presence of a positive spillover of the program. For example, some treated *ejidos* in the area have been able to successfully develop ecotourism projects in parallel with the enrollment of their forest program [38]. However, asserting the existence, drivers and consequences of these behavioral and collective responses with full confidence would require extensive ethnographic work at community level.

Overall then, given that deforestation has proceeded apace in non-PES and a minority of PES targeted areas during the study period, we think that the PES program has been insufficient to halt deforestation at municipal level. This outcome is explained by the existence of incentives to convert forest lands to more profitable land uses such as oil palm cultivation [6,37], resource management competing interests and forest governance failures at *ejido* level, and lack of enforcement of the PES program by CONAFOR. The long-term permanence of current high additionality levels in the studied municipalities is likely to be dependent upon sustaining payments over time and keeping PES targeted areas sufficiently large, in combination with alternative conservation strategies ranging from improved command and control approaches to investment into more diversified forest agricultural economies. A comprehensive conservation and development strategy appears as the necessary next step to take into account all together conservation priority zones, biodiversity corridors and agricultural development hot spots [18].

## Supporting Information

S1 File Sources of data used. To perform this analysis, we have used databases provided by different organizations. We provide information on the availability for each of these sources.
(DOC)

S2 File Data generated by the authors. (ZIP)
