# Supplementary material for: How Effective Are Biodiversity Conservation Payments in Mexico?
Source: PLoS One. 2015 Mar 25;10(3):e0119881. doi: 10.1371/journal.pone.0119881 (PMC4373862; doi:10.1371/journal.pone.0119881)
Supplement: S1 File — To perform this analysis, we have used databases provided by different organizations. We provide information on the availability for each of these sources. (DOC) [file pone.0119881.s001.doc]

**S1 File. Sources of data used**

*Database available online:*

Census data at village level are available in the website of the Mexican National Institute of Statistics and Geography (INEGI): <http://www.inegi.org.mx/> .

Administrative data for *ejidos* and communities are published in the online database of the Mexican National Agricultural Registry (RAN): <http://phina.ran.gob.mx/> .

List of the forest owners participating to PES programs are published each year by the Mexican National Commission of Forests (CONAFOR): <http://www.conafor.gob.mx/> .

*Geo-referenced information available online:*

Digital elevation model (DEM), municipal and state boundaries as well as localities position are available in the website of the Mexican National Institute of Statistics and Geography (INEGI): <http://www.inegi.org.mx/> .

PES program and Special program for Lacandon rainforest eligibility areas are published each year by the Mexican National Commission of Forests (CONAFOR): <http://www.conafor.gob.mx/>.

Protected areas are available in the online database of the Mexican National Commission for Knowledge and Use of Biodiversity (CONABIO): <http://www.conabio.gob.mx/informacion/gis/> .

Deforestation risk index is published by National Institute of Ecology and Climate Change (INECC) and is available at: <http://www.inecc.gob.mx/irdef-eng> .

International boundaries are published by the website Global Administrative Areas (GADM): <http://www.gadm.org/> .

*Geo-referenced information available with restriction:*

PES polygons can be available upon request with Sofia Cortina, leader of Forest Environmental Services unit (gerencia de servicios ambientales del bosque) at CONAFOR: [scortina@conafor.gob.mx](mailto:scortina@conafor.gob.mx)

Geomorphologic map can be obtained from José Gerardo Garcia-Gil at [ggarcia@ecosur.mx](mailto:ggarcia@ecosur.mx) .

*Ejido* boundaries have not been officially published by an organization, so we have elaborated this map in coordination with local actors. Therefore, we cannot provide this map.

Land use and land use change maps have been generated from Spot 5 satellite images bought from the French National Centre for Space Studies (CNES) through the Incentive for the Scientific use of Images from the Spot system (ISIS) program and through the ERMEX project. We do not have the right to share the satellite images.

*Data generated by the authors*

In the S2 File, we provide the information we have generated and used for the analysis.

“datapsa.cvs” is the statistical database used to perform the analysis.

“variable_description.xls” is the description of each variable

The folder “grid” is the georeferenced database corresponding to the statistical database. It can be open using the software Arcgmap®.

The folder “land use maps” contains the classified land use maps for the year 2007 and 2013. The document “landuseclasses.xls” describe the land use classes we have defined.
